# Supplementary figures and images for: Developmental changes and metabolic reprogramming during establishment of infection and progression of Trypanosoma brucei brucei through its insect host
Source: PLoS Negl Trop Dis. 2021 Sep 20;15(9):e0009504. doi: 10.1371/journal.pntd.0009504 (PMC8483307; doi:10.1371/journal.pntd.0009504)

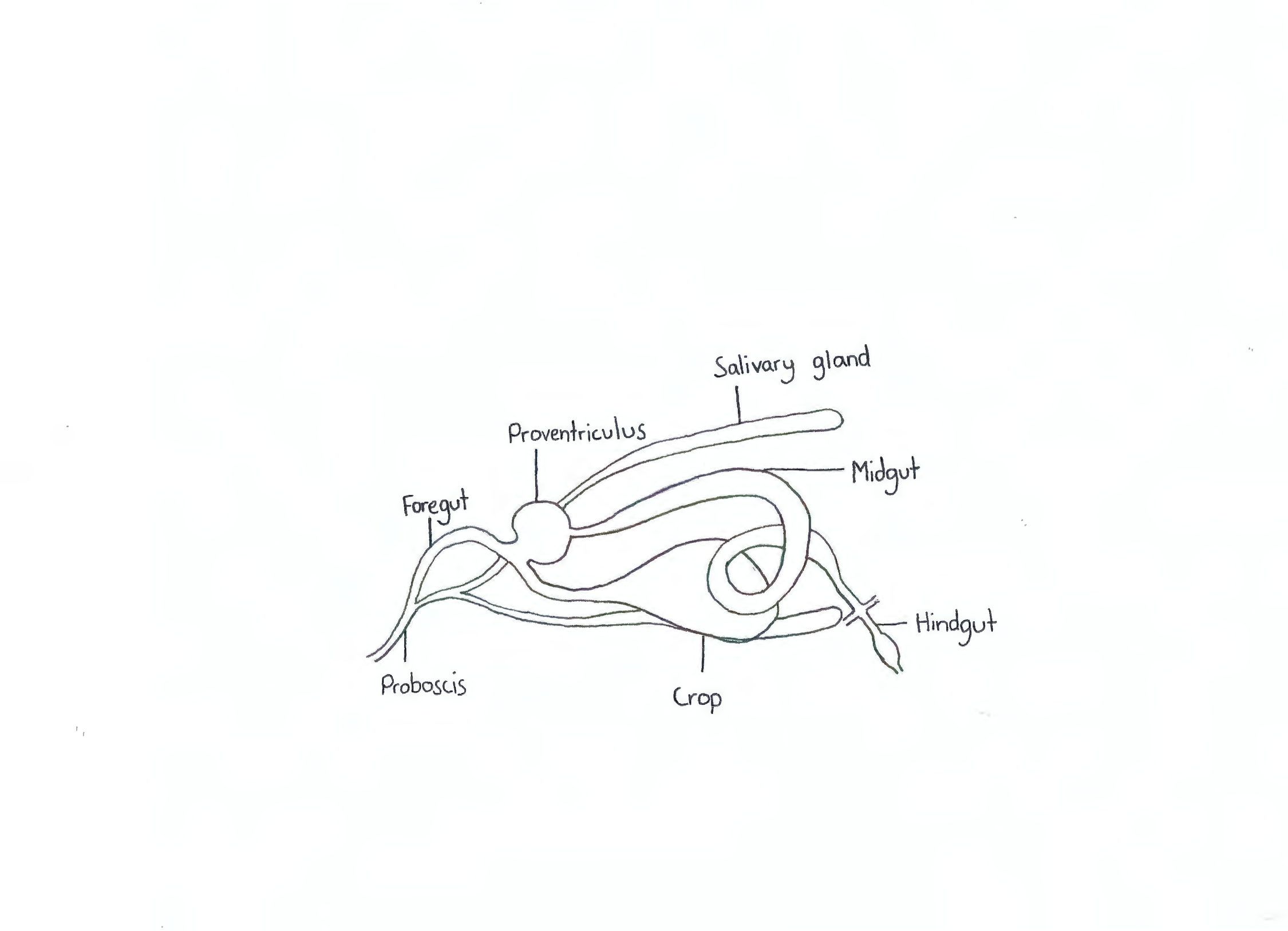

Supplement: S1 Fig — (TIF) [file pntd.0009504.s001.tif]

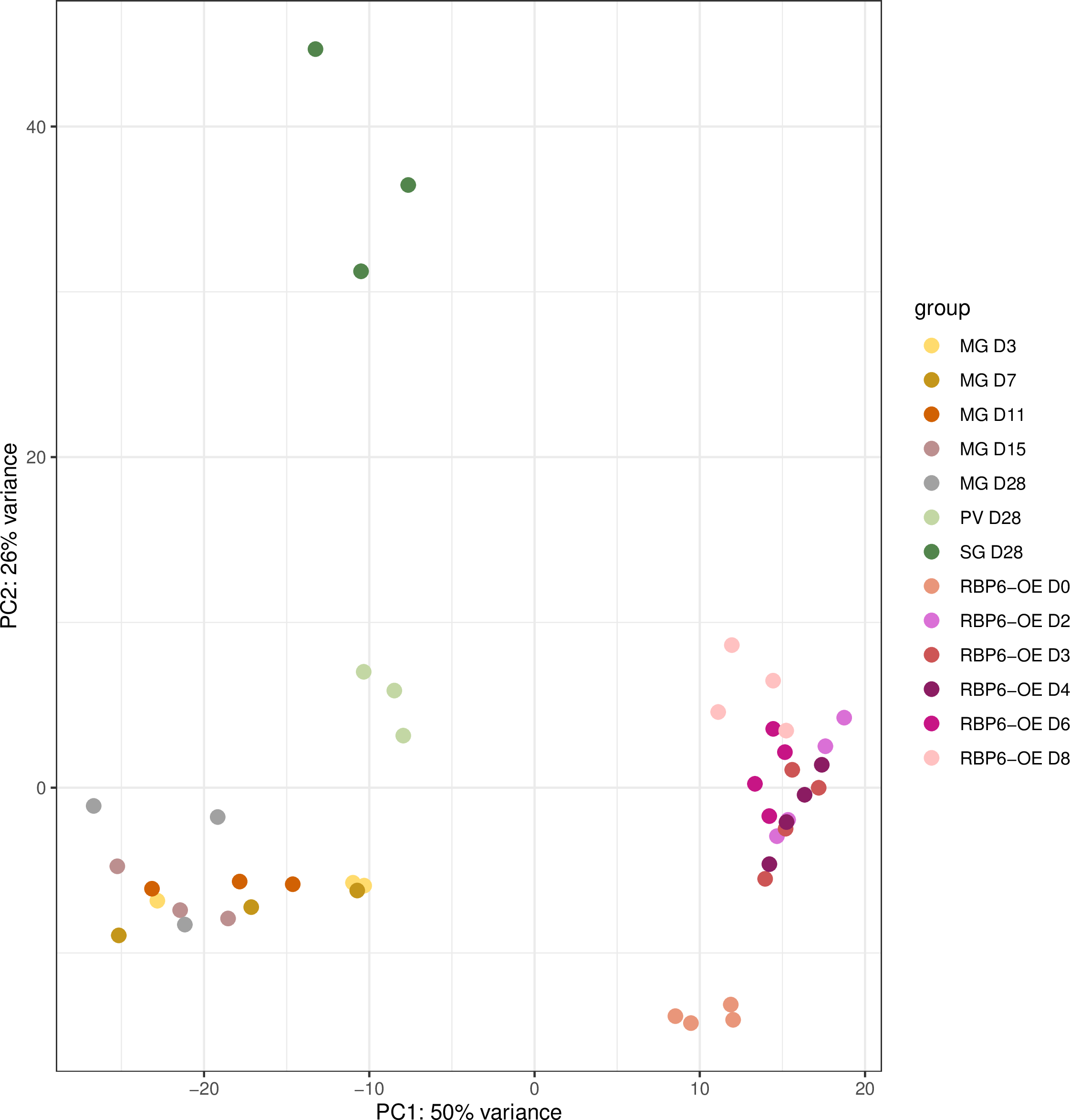

Supplement: S2 Fig — (TIF) [file pntd.0009504.s002.tif]

## Slide 1
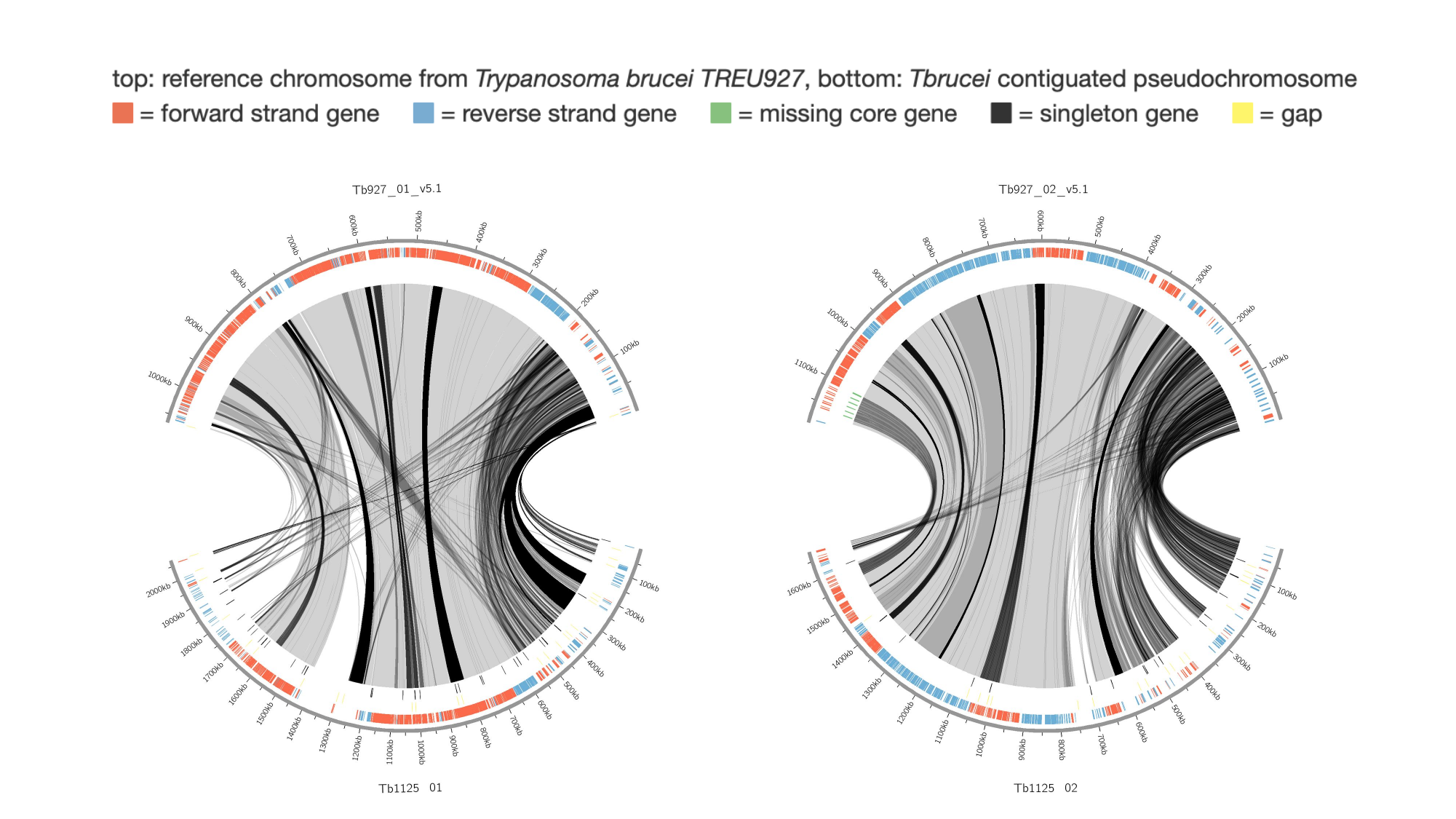

## Slide 2
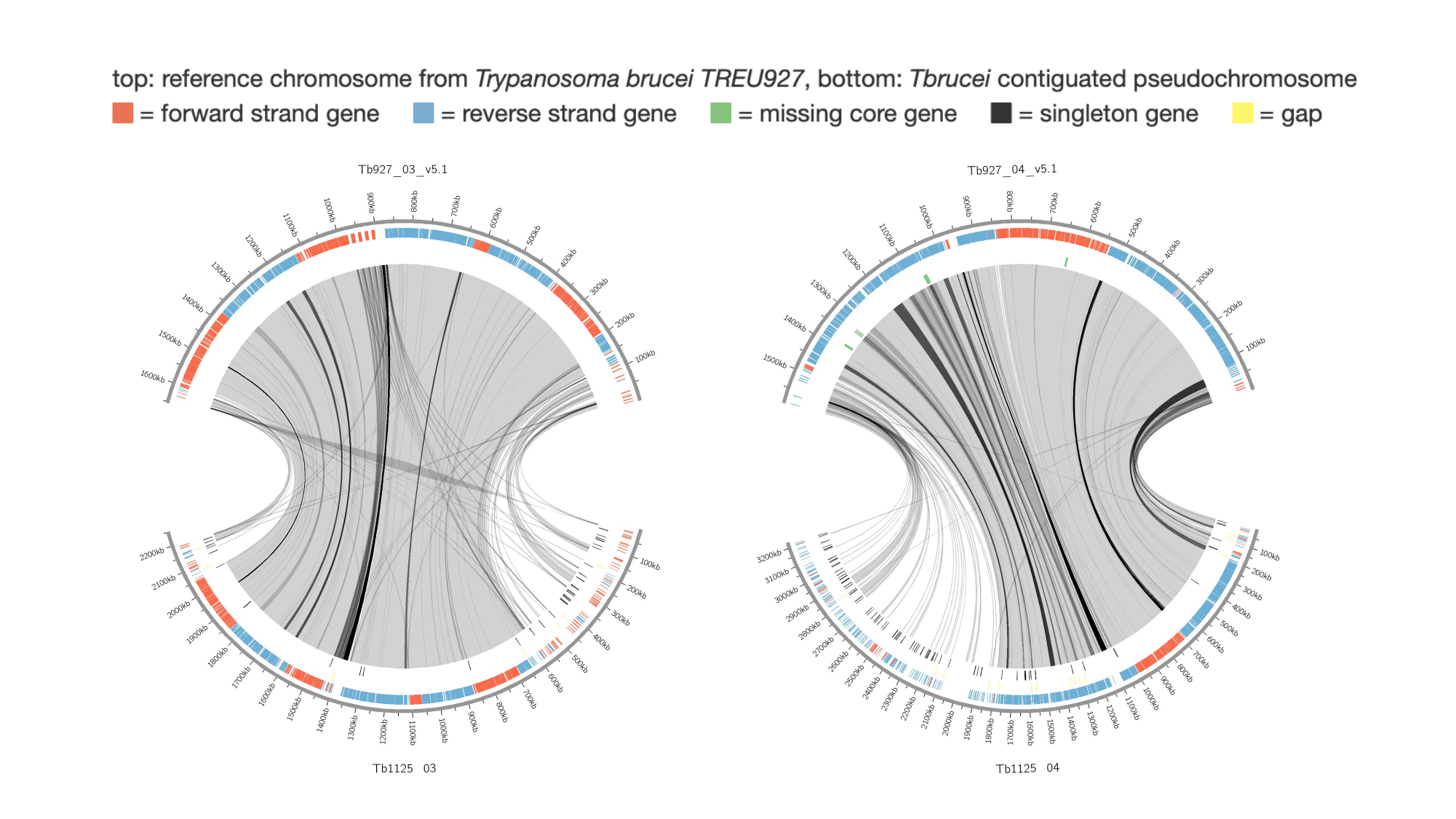

## Slide 3
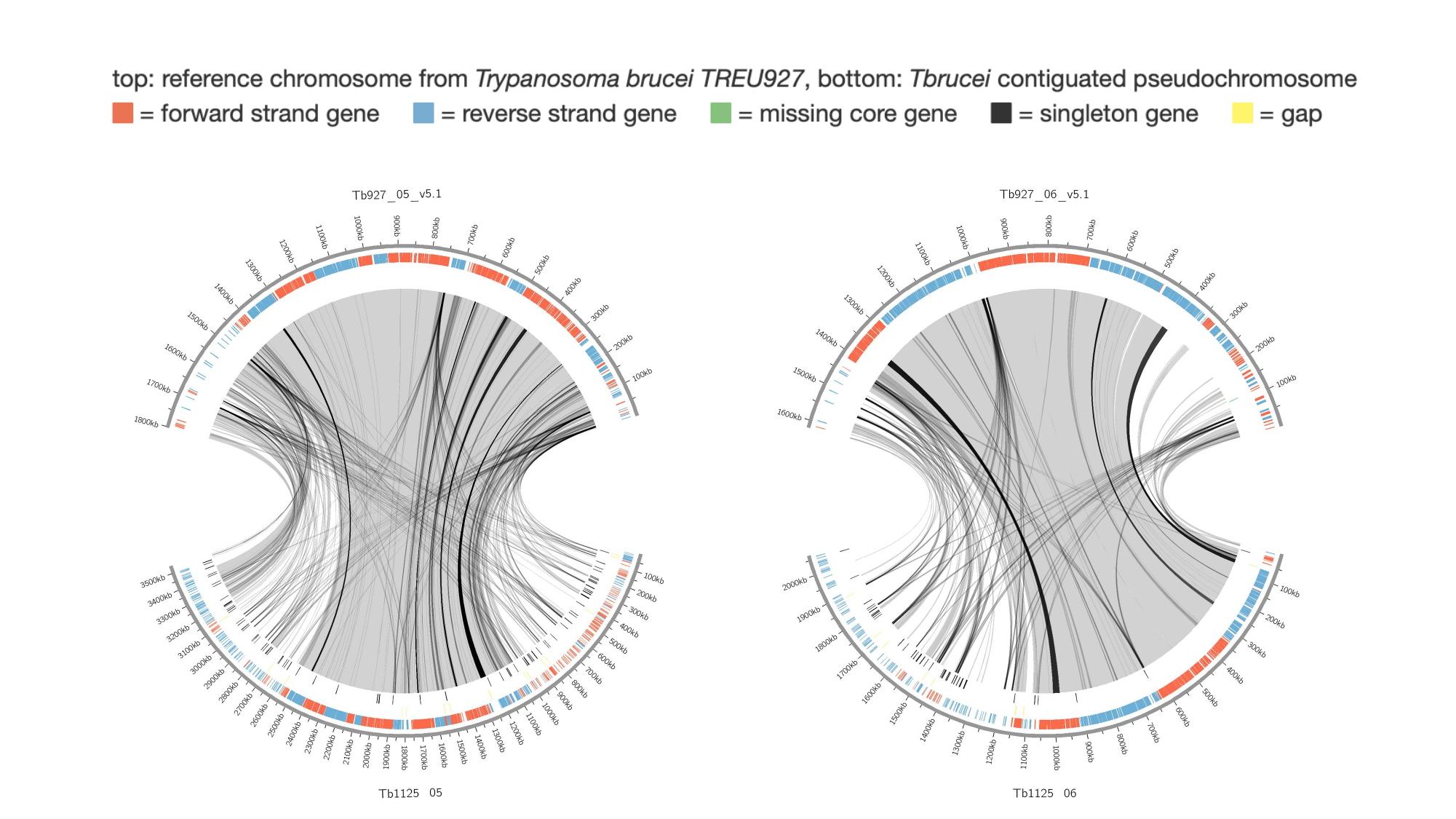

## Slide 4
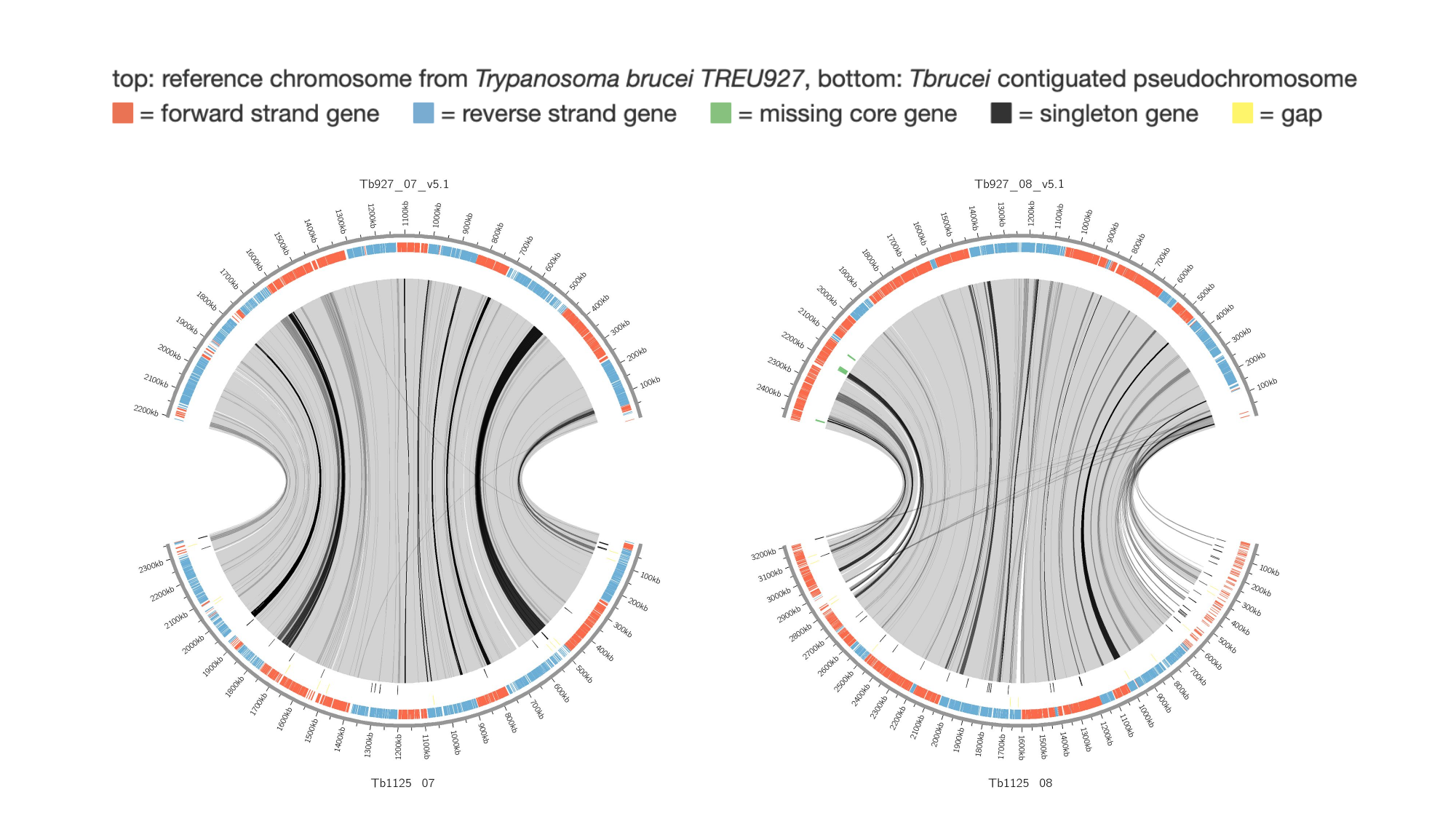

## Slide 5
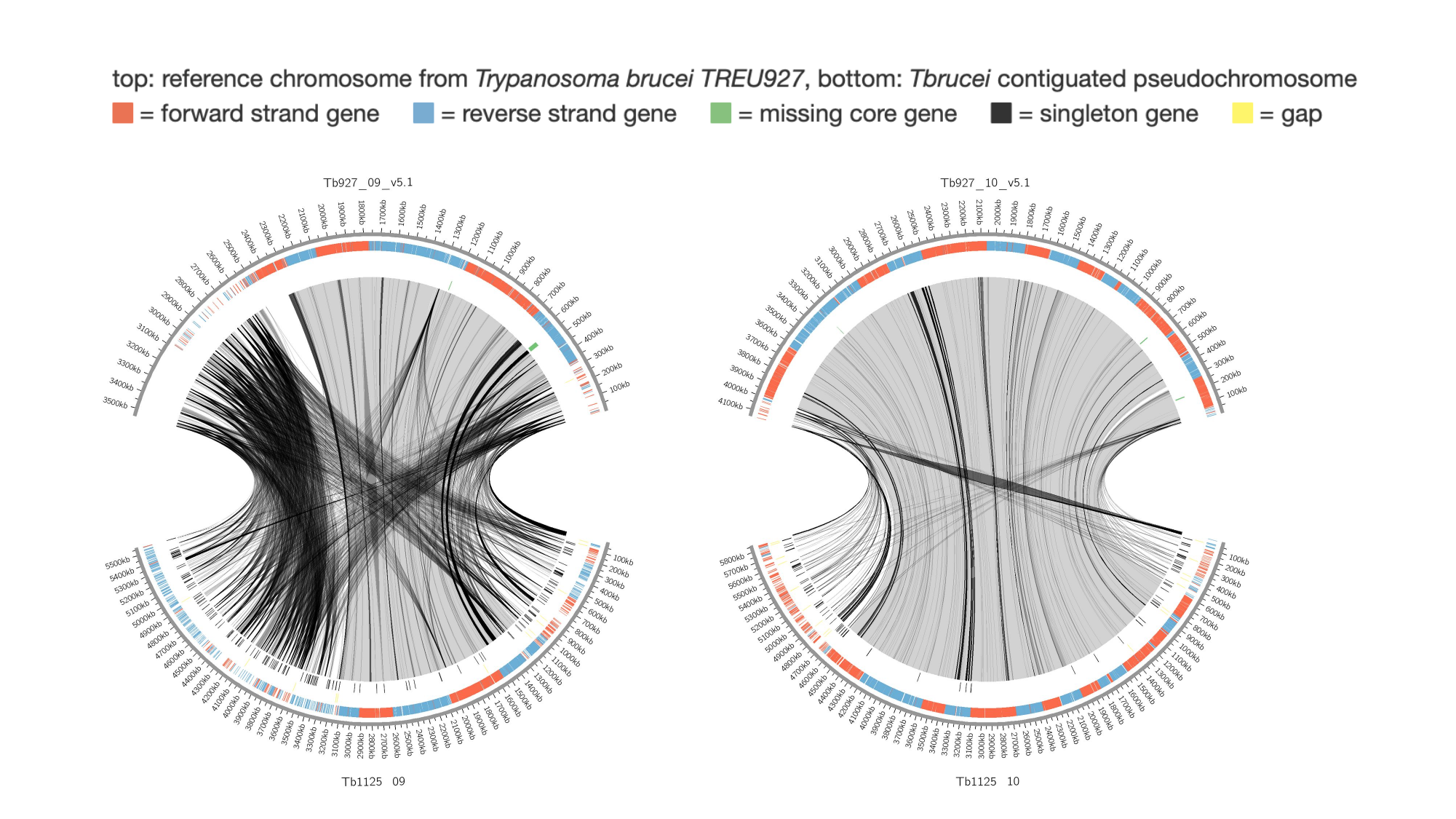

## Slide 6
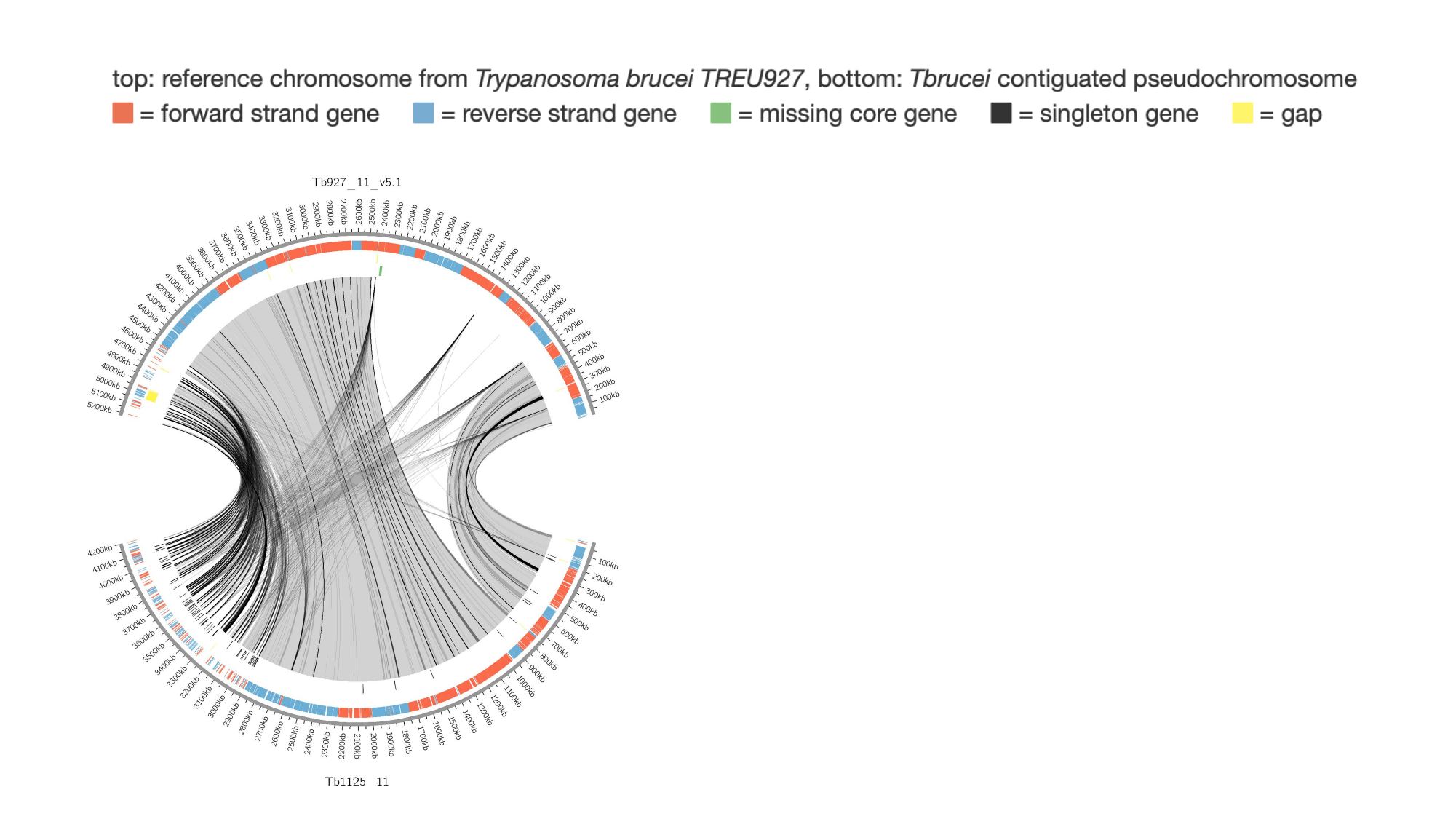

Supplement: S1 File — (PPTX) [file pntd.0009504.s008.pptx]
