# Supplementary material for: Developmental changes and metabolic reprogramming during establishment of infection and progression of Trypanosoma brucei brucei through its insect host
Source: PLoS Negl Trop Dis. 2021 Sep 20;15(9):e0009504. doi: 10.1371/journal.pntd.0009504 (PMC8483307; doi:10.1371/journal.pntd.0009504)
Supplement: S5 File — (DOCX) [file pntd.0009504.s012.docx]

**Metacyclic variant surface glycoprotein (mVSG) transcripts**

>mVSG1 | length=1560 |

TATTAGAACAGTTTCTGTACTATATTGCCTTTTCCTGACGCTAGAATTGGCAAACGCAAATGCCCAAGACATCAAGGCTATGCTCGCTAGGCTTTTTTGTCCTAGCGTTGCTTGCAGAAAGCTCACGTACGGCCACAACGTACACGATCTGCTCGACGCCGTGTAAATGCAGCAAACGACTCGGCAAAGCATCGACATTCTATGCTCAGAAGTTTGAAACCAACGTCGGCAAGCTTGTAAAGATGCAGACAGATTTAACAAAACTGCTTTTAGCGGCGACAGCAGCCGACGTAGCAACTGCAAAAACCGCTCTACCGACTCTGGCAGCGGCGGGAAAAGTAATTCAGGACTGCCAGGCAGCTGTCACAAGACAGCTTGCGGCACTGAAAACGGGCCTACCAAAGATAGCAAACGCCTCAGCGAAACTTGCAGCTCTAGCAAGACGGCAAGCCACAAAGACGACAGTCAAACTGACTCCAAAAGGCGGGACCAGCAACTACAAAGACACTTCGTTCGCGAACCCTCCCGTAGGCATAACCAGCGACGACAGCTGCGGCCATGAAACAGGCGACGGCGAAACCGACTACGACGACAACGAAGCAGACGAAAAAAATGCGATACTGGAACCCCAAGAATATCACACAGTAACTGTAACTTGCAGATCAACAGGCACAACAAACTGCAACAGCGCAGGACAAACGCAGGACGACGGCTACATCCAATTCGAGCTCACATCGGCCATAAAAGAAGAAACATCCAAGCCAACTACACGGTGGAATGCAGCCAACACCGACAGCGATGTAATAATTCACGGAGCGGTAAATGTGACGCAGGGAACCAAAGAGCCGACGGAAGCTGCCCTGAAGGAGCTCAAACAGGCAGCGCAAAACACGGCGTGCGACAAAAAGCTAACAGAATACGCAACAGTTTCAGCATCGCCTTTGTTCAGACGCCAGGCAATCCGTAGTTTGCTCAACCAAGACGCCGACGCACAGGATTTCACAACACCCCCAGACAAGCTAACCGGCGCCCTAACGGCAGCTTACGGCGCAGGCGGAATGGACTACAAAAAAAAGTTGTGGGAAGCCATAGACAACCTTAAACCAGCGATAACAAAGAACAAAGAGCGAGCCGAACTGGACATTAAAGAAAACACACCTCTTGAGCAACTCACTGAAGCCCTTGCACGGCAGATAGGCGAAGCTAACTCGAAGGCATCACAAACAACTAAAAATAACAAAAATGCAAACGATCCAAGCAAGTCAGATGCAGCAGACAAAAAAGAGGAAAAGAAAGACGGGGATCAAAAAGACGAGGAATGCAAAGCCACTGAAGAAGGTAAATGTGACAAGACAAAATGCGATTGGAACGCTGAGAAGAAAGAGTGCAAAGTTAAGGAGGGCGCGGCTGTTATTTCTGCTGTAATTAAAGCCCCTCTTTTGCTTGCGTTTTTGCTTTTCTAATTCCCCCTCTTTTTCTTGCTAAAATTTCCTTGCTATAATTTTTGCTACTTGAAAACTTTCTGATATATTTTAACACGCAAGTTACCCGAAAAAAAAA

>mVSG2 | length=1623 |

TATTAGAACAGTTTCTGTACTATATTGCTACTGGAAGAAGTCAAAATTAGTAACGAAAACAGCAGCC GCAACTATTTCTCCAAAAAACAGCCTTTTCCTGACGCTAGAATTGGCAAACGCAAATGCCCAAGACATCAAGGCTATGCTCGCTAGGCTTTTTTGTCCTAGCGTTGCTTGCAGAAAGCTCACGTACGGCCACAACGTACACGATCTGCTCGACGCCGTGTAAATGCAGCAAACGACTCGGCAAAGCATCGACATTCTATGCTCAGAAGTTTGAAACCAACGTCGGCAAGCTTGTAAAGATGCAGACAGATTTAACAAAACTGCTTTTAGCGGCGACAGCAGCCGACGTAGCAACTGCAAAAACCGCTCTACCGACTCTGGCAGCGGCGGGAAAAGTAATTCAGGACTGCCAGGCAGCTGTCACAAGACAGCTTGCGGCACTGAAAACGGGCCTACCAAAGATAGCAAACGCCTCAGCGAAACTTGCAGCTCTAGCAAGACGGCAAGCCACAAAGACGACAGTCAAACTGACTCCAAAAGGCGGGACCAGCAACTACAAAGACACTTCGTTCGCGAACCCTCCCGTAGGCATAACCAGCGACGACAGCTGCGGCCATGAAACAGGCGACGGCGAAACCGACTACGACGACAACGAAGCAGACGAAAAAAATGCGATACTGGAACCCCAAGAATATCACACAGTAACTGTAACTTGCAGATCAACAGGCACAACAAACTGCAACAGCGCAGGACAAACGCAGGACGACGGCTACATCCAATTCGAGCTCACATCGGCCATAAAAGAAGAAACATCCAAGCCAACTACACGGTGGAATGCAGCCAACACCGACAGCGATGTAATAATTCACGGAGCGGTAAATGTGACGCAGGGAACCAAAGAGCCGACGGAAGCTGCCCTGAAGGAGCTCAAACAGGCAGCGCAAAACACGGCGTGCGACAAAAAGCTAACAGAATACGCAACAGTTTCAGCATCGCCTTTGTTCAGACGCCAGGCAATCCGTAGTTTGCTCAACCAAGACGCCGACGCACAGGATTTCACAACACCCCCAGACAAGCTAACCGGCGCCCTAACGGCAGCTTACGGCGCAGGCGGAATGGACTACAAAAAAAAGTTGTGGGAAGCCATAGACAACCTTAAACCAGCGATAACAAAGAACAAAGAGCGAGCCGAACTGGACATTAAAGAAAACACACCTCTTGAGCAACTCACTGAAGCCCTTGCACGGCAGATAGGCGAAGCTAACTCGAAGGCATCACAAACAACTAAAAATAACAAAAATGCAAACGATCCAAGCAAGTCAGATGCAGCAGACAAAAAAGAGGAAAAGAAAGACGGGGATCAAAAAGACGAGGAATGCAAAGCCACTGAAGAAGGTAAATGTGACAAGACAAAATGCGATTGGAACGCTGAGAAGAAAGAGTGCAAAGTTAAGGAGGGCGCGGCTGTTATTTCTGCTGTAATTAAAGCCCCTCTTTTGCTTGCGTTTTTGCTTTTCTAATTCCCCCTCTTTTTCTTGCTAAAATTTCCTTGCTATAATTTTTGCTACTTGAAAACTTTCTGATATATTTTAACACGCAAGTTACCCGAAAAAAAAA

>mVSG3 | length=1608 | **Tbb1125VSG-4862**

TATTATTAGAACAGTTTCTGTACTATATTGTAAGGCAAGCAAAAGGCAAAAAGCAGCCGAAAGCTAAGCATCTCCACGCGCCAATACAGTCCCGTGTTTGCGAGCGTCCCGGCCCGCAGCAGCGGCACCAGCCGTGCAACAAAAAATAGTCTTTAAGGCAACGCTGTTAAGATCGGCACAGATACAATGAGAGGTAAAGTGTATGCTTTAGCCTTGTTGCTAGCCACCCACGACTCTGCAGAAGCAACTCCCAACGGCATAAAAGCAGGGGCGATAAACAGGCTTTGCTCATTGTCGGACAACCTGAAAGCCTACACAACATACACAAAAACAGCAATAGCAACGGCACTAACGGCAGTAAATGATGTACAGACTGTCAAGCAAAAGCTAATCCTCTTGGTTCTGCACACCAAGAAGCTCCCGACGCAAAACGCCCCACTCATTCTCAGTTTTATGGAGCGGACGATATCTCAAGCTATTTTGGACCTTAAACAAAATGCCCCAACGGCAGTGTTGGCGGCGGCGGCCGCAGCACTTAGCAGTGGCAGAATCGATGAGTACACTCAGCTACTTTACAACGCCAACACAAACCAGCCGGGCCTAGACAAGTACTGCGTCGCCAAAGATACTGGGGCGAGCAAAGCCGACCCAACAGACTTAGGCAGTTGCATAAGCGAAGGCAAGTGGAAAACCAAGATAACCGACGTGCCCAATACCGTGGCTCCAAGCTACGCAACAAGCGTCCCGAACCAAGCAGATGCGCAATCCGTAACTGCACACACCGGCCAAGACAACGGCTGCATGCTGCTTTCCTCCGCCAGCGGGGATGGCTTTGGTGTAACATCCCACGCGGGCAAGGATATACTAGTCATGGGGGGCATATTCAAACTAGGGACGACCGCCCTGGCGGCATCGCCTTTCAAGGATGTTTCGGCGACGGCGGCGCCTAACACACCGTTAGAACAGCTAAAAGCGATAGACCACAGCCAATTCCCAAAGATTGCTGGACCGGCAGTAACGATACTTAAGAAGCTAGCCAGCCTAGGCAAAGGTGCAGACTTCGATATACCCGACCAAACGATACAGAAGGCCGACTATGGACTCGGAGCTGAAGGCACCTTACAGATAGCCAGCAGCGATTTTAAGAAGATAAATACAGCGCTGCAGCAATTCGAGGCGAGCGCATCCGATGGCCTGCAAAGGTTGCTAACCCAGCTTCCCAGCGCTTTGCTTAGCCATGCGGCCATGAACTGCACCGTTCAATCATCAGCAGGCGGGGAACCGAAAACAACCGCGGATAGAAGTGACTGCAATTCGCACGCGGAGGAAGAAGCGTGCCGGAAAGCTAATTGCAACTTTGATGGCTCCAAAAAGCCCAAGTGCTTTGCTAAACCAAGTGAAAATCAAAGCGATAAGCAAGATGGAGAAAGCCGTAAAAAAGAGCAAAACGCCACAGGGAGCAATTCTTTTACAATTAAGAAGGCCCCTCTTTTGCTTGCATTTTTGCTGTTTTAATTTTTTTCCCCCTCAACTTTTTAAGAAACTTTGCTAAAAATTTCTTGCTACTTGAAAACTTTCTGATATATTTTAACACCTAAAAAAAAAAAA

>mVSG4 | length=1473 |

ATTATTAGAACAGTTTCTGTACTATATTGTTATTTTCAACACAGTCTGACTAAAAAGCAAAAAGCAAGAAAGAATCAGCGACGATGGCGACGCTGCGGGCGGTTTTATCGACAACTTTAATTTGTTTCGTATTCTCCACTGCCAGGGGAGCAAAAGAAACACTTGACGCGACAAACCATATAAAGCCGATCTGCAAGACAAGTGGTCAGTTGAAGGCACTAGAACTCTATATCGCAAGCAAACTTGAAACCGGTAGGCAAGCGGCCAATGGGCTGAGCGCAAGGCGAGCGCCCTTTACACTCTACGCAATGTCAACAGAAAGTCAGGCGGACAAAACAAACTGTCGCCTAGTTGCAACTTATATCGAAAAAAAAGCTGCCAGCGTCTTAACCGATATGGCGGAAACATTCGTAGCAGCTTTAAAGGCAACAGGTGCAGCTCATTATGCAGCAGGAGTCATAGACGGTGGCATCGAAACATTGGCAAGCGAGTCAAGCAGCGGCGAGCACTGCATCACCAAAGCAGACGGAACGACAAAAGCTGACAAAAGCGATCTTGCAGGGTGCGGAACTGCGGACGAAGATTATACACAATACAAAAACGACTGGGAAACAAAAACAGCTGGACAAACGCTAAGCGCGATAACGACAGAAGCGGATATAACAGTCGGCGGCTCAAGCAATAAGTGCGCGCTTTGGACGGGCGCATCAAACTTACACAGCCAAACAGCGCAAACAGTAGTCTGGGCAGGAGGCACTATAGCCTTCGCGGCTGGCAGCGACGACACAGTCCAGCGTCGCCAGATAACAAAGTCAAACACACCAAAGATTTTCCCGGCCATCAAGCTGATAGACGACCTGAGAAACAAAATCTCGACAGCCAGCTGGACCGACCCAATGGAAGCATTTTTCAGCAAAAGCGGAGATGACAGCGCCGAAGCACTAGCGTTAGCGACGGAAATCAGTAACAGCCTCAAAAACAAACCGACAGACGCTGAGCCCAATGTAAGCAGCGTTAAGGAGACGTTACAGGCAGTCAAAAAAGCGAGTAAGCAAGATCCAATCAAGTCGCAGCTTACAAAGACGCCACTTCAGAAACAAGCGGAAATTCAGCTTACAATCAACACATGTCCAACCTGCGAAATAAAACTAGCTGCAGCCACCTCCTCAACAACCAGTGCAAGCACAGGCACAGCAGCAGATTGCAAATCCAAGAAGGGAGATGAATGCAAAGGCGAATGCGAGTTGGATGGGGAATCTTGCAAGCCGAAAAAGCAAGCAGAGGAAGTGAAAAAAGAGAATGAAAAAACCACAAACGCCACAGGCAGCAATTCTTTTGTCATTAACAAGGCTCCTCTTTTGCTAGCATTTTTGCTTTTTTAAGTTTTTCCCCTCTTTTTCTTAAAAAAAATTTTGCTACTTGAAAAACTTTCTGATATATTTTAACACCTAAGTTACCCGAAAAAAAAAAAAA

>mVSG5 | length=1727 |

GAACAGTTTCTGTACTATATTGTTCAAATCTTTCTATAACAACTGAAACAACAACTCTTTGCCAAAGCAGAAGCAACACTGAGAACTCCACAGATGATAGGAAAAGCCTTTATTATTTTATCTTTACTTAACGAGCTGCCAAGCCCGACGGCAGCACAAGCGGCCGAAGGTGGTGCCCTCGGAAAAGACGTATGGCTACCTCTCGCTAAATTCACGGCGACGGCCGCGAAAATCCCAGGCAGGGCGGCAAAGCTGCTTCAAGACAGGTCGGCCCAAATAGTTAACCTTATGAAACTCCAAGTTCAGGCAGACATATGCCTCAACAAAGCAGCGTCAGAGGTGAGCGCACTTGGGTGGCAGGCGCTCGCTGTTGCAATAGCAGCAGACATCGGCAGCCTGCAAAGCTTGCAACAGCAGAGGAGTGAAGAGGCAATAGCGGCCGCGGCAGCTGCCGAATTCGCTCGGGGCCACGCAGCGGAATTCTTCAAAGTAGCTGCGGCAGTCCAAAGCGCCGCCAATAGCGGCTGCCTGACAACAAACAATAAAGGTGGCGCAGCCGGCAGCGTGATAAACGGATTCTCGACACTCGGCACCGCGGAGCAGCCAGCAATCGGTGCTACATCGACGGCTCACGTCGGCGACGACATAACGGCGATAACAACAACAGGGTTCAGCGACCTAGCAGCAACAGACGGCATACGCACCGACTCACTAACAGCGGACACAAACTGCGTTCTTTTCAAGGGAGGCAGCGCTGGACCGCTAACGACAGCAAACTTCGGCCAATCGATCCCTTTCGCAGGCGGCTATCTAACAAGGAACCCGACAGCCAACACAGCCAGCAGCGCCGACGGTACGGACTTTGTAACCAACCCCGGCGACGGCAAGATAGCAGGCATAAAAGTCTATAGGGACGCCCACGCCGCCGCAGCGAAAATACGCACAGCAGCAACCTTCGGCTCGAGCTTCACCGACTTCAAGAAGCTAGATCAGGCTAAGAAGTCAGTCCATTTGCGCACAGCAGTAAAAAACATAATTCTCGGCAAACCTGACGGATCCGTAGCCGACCTTTCCGACGAGATAGACACAAAGATAAACCAGGTATTCGGCGAAGACCAAGCAACATTCCACAGCAGGTTTTGGGATCAACTAACAAAAGTAAAAGTGGAAAAGGCGGCGAGTGGGCAAGACGAAACGACCCTCGATGCAATCACTTCTTTTGCAGCCTTAAGCCGAGCTCGGACTTATTACTCCACGAAAGTGATTAAAGGTTTGAGAGATAAGATATCCTCACTAGAAATTAAAAATTCCAAAACGGAAGTTAAAGTCACTGACGCCGACTGCAACAAACACCAATCAAAAGACAAATGCGCAGCCCCATGCAAATGGAACGAGAATACCACTGACATAAACAAAAAATGCTCATTAGATCCCGTAAAAGCGACAGAACAGCAAGCAGCCCAGACAGCAGGAGCAGGAGAAGGAGCTGCAGGAACAACAACAGATAAATGCAAAGATAAGAAAAAGGATGACTGCAAATCTCCGGACTGCAAATGGGAGGGTGAAACTTGCAAAGATTCCTCTATTCTCCTAAACAAACAATTCGCCCTAATGGTTTCTGCAGCCTTTGTGGCCTTGCTTTTTTAATTTTTTCCCCCTCTTTTTCTTAAAGAATTTTTGCTACTTTAAAAACTTCTGATATATTTTAACACCTAAAACCAACCGAG

>mVSG6 | length=1596 | **Tbb1125VSG-39**3

TATTAGAACAGTTTCTGTACTATATTGTAGGTGACACGACAACAGAAAACAAAACAAACCATTTAGAAGTCTAAGTAAAGGATGCGCCATACCGGCATCAGTACCTTATGTGTGTTCTTGCTGGCATTGACGAATAGCCGCGCTGCCAACCACCACCCTCTCAAGCTCAACGCGTGGAAGCCAGCGTGCGAGCTAGCAGGACAGCTAAAATCGGTGACAAGTGTAGCGCTAACAACAATTCACAGGCACCAGCAGGAAAGCGATGCCAGCCGCAAAACCGCGTACGCAGTCCTGCTTTACGCGGCCGCAAACAAAGACGCTGCTGCAGACATTGCCGCCGAGGGCCTGGCAACGGCGCTTTTCCAGCACAGCGAGGCTCAACAAGCGACTGCAGCTAGCAGCATGCGCATCGGCCTTACGGCGACCCAGTCGGCAGCGCAGCTCAGCGGCGCCATAACGGCCACGATAGATTTCCTCGCAGCAGCCAGCCACGACGCTGTATATTGCCTAGGCAACTCGGCCGGATCTGCAGACGATGCAGCAAATCGGCGTAGGTACGGATGTTCATACGCACTAGCCGAGCCGACGGCCGACGCCCAGCTGCTTCGAAGCCCGGCCGTGGACTCGAAAGGTTTTGCCGAACTAAGCGATGTAGTCGACGGGTCGACGGTCGACTCAAGCGCTCAAGACAAATGCATTGTAACAGAGCATGGTTCAGACCACACAAAACTGCTCAACGACGCTGGGCACAACAACAAGCTGGTCGGTGGCCTCTTTGACTTCACGACCGGCAGGCAAGCGAAGAGGTCAGGCTACGCGACGGTGGCGCCAAAAGGCGAGAGGGCGACCCAAGAAGTTCAAGCTACTGCTTTCCACGACTACGCCGAGCTAAGGGCAGCGTACAAGTCGAAGGCCACAACGGATCCAGTCGAACTGATAGCAGCAGTAGCCGCAAGCGATTACCTAAAGCCCGCGGTTGCTGAATACCTAGCAAGCACATCAGGAGCAACCAAGCCGACAAACCACGAAGCAATGGCCTCGGCCATAATTGACAAACACTACAAAAACAATGGCGACGGCATCAAAAACCTCTGGGAGAGGATTAAAAACACACAAGTGCTCGATATAACAAAGGCGAACGGCATTAAAACCAAAATCGTACAAATAGGTACCGCCAGTACCCTGCTGCGAACACTAGCGCTTTACAACCGGGAGGCACTCGCTAAGATTTCCAAACTAGAAGCCGCAGCAGCGGAGAAACCGACGGCCAAAACAACAAAAGAAAAGGCACCAACGGAGGAACAATGCATGCACCACGAAACTACAGGAACCTGCCAAAACGAGGGTTGTGAATTTGATGAAACCAAAACGCCAAGATGCTTTCCGAAACCGAGTGAGACCAAAGAAGAGAAAAAAGACAAGCAAGATGGTAAAACGAACACCACAGCAAACGATTCTATTGTCATTAACAAAGCCCCTCTTTTGCTTGCATTTTTTCTTTTTTAAATTTCCCCCTCCCTTTTTAAAAACTTTGCAACTTGAAAAAACTTTCTGATATATTTTAACACCTAAAAGTTTCCACCGACAAAAAAAAAA

>mVSG7 | length=1554 | **Tbb1125VSG-4959**

ATTAGAACAGTTTCTGTACTATATTGATAATATTAGAAAAAGCAAAAAACGGCAGACATGAAGCAACACCTAGTTTTCGCTGCTGCTGCGGCGATAATCAGCTTAGCGCCGGCGGCGCACGTCGAAGCAGCAGCCGGCGATGCACTAAACCACGCAGCATGGTCCAAACTCTGCGACATAACGAGGGACCTAGATAACCTACCGTCGAACGAGCTAGCGTCGATAGCGGCCGAGCAAGTAAACGTTGGCGACCTGACGAAGCTTCAGCAACAGCTAGCGCTCTACAGAGTGCTAAATACCGACAAGGCGGCCACAACCGCAGAACAGGTGTTTGCAACCTTTTTATCAAGAAAGATAAGCGGCGCAGCGATAAGCGAGACTAAGCTAAAGGAGGCACTGCAAAGCACAGCAGATGCTGCTTTTTTGCACGGCCAATTAGCGGAGTGGCTAGCAACAGCCAGCTCTATCGGCGGCAACACGGCGGGCTGCCTAGGGGCGGCCGGCGGAGCCGACACAGCGGACAAAGCGACGATCACCCAGGCCCCGTACGCCTGCAAGCTGACAACGCAAATGGCAAGCGCCAAGCTAACGACGCCGGCAAGCATAGACGCCAACGGCTACACAGGCTTCACTGCGATACCAAACGTCGAAACAAGCAACGCCGTAACGCAAAAAAAGTGCGCATACTCCCAGCACGGCGCAAGCGGGCTGGGCGGAGCTCAGGACACGACAACCAGCATAGCATTCGCAGGTGGCGCATTCATCCTAACAGACAACGCCCTAACACGCAGCAACTGGGCAGCGACGTCTCAAGCGGGGACGCTCCCGCATGAAAAAGCTTTCAAAGCTCTGAAGAGGCAGCACAAAAGTCTGCCGAAAGCAAACTACACGAAAATAACTGATCTAAAGCAAGACAGCGACTTTAGAAGCGCCGCGATCGCGATAATATACGGGGATCAGGACAGTTCACAACTGGAAAACAAACTGACAGACTTGTTTGGCGCCAGCGACAACAGCTTTGAAGAAAAATTCTGGAAAAACGTTCGCGAAGCAAAAATAGACGGCGGAAAATTCGGCGCAAAAGAAGCGACAACCATCCAGGCCTTAGAAGACCCAATAAAACTCAGCAAAGCGTTTTATTATTACGCCAAGCAAGGGACGGAGCAGTTAAAAACACTGGAGGCGAGAGCGGCAGCAACCAACGACCTTCCGAAGCCAACGGAGGAACTTTGCAATGCGAAAAACGACGAGCCCAAAGCATGCAACGAAGCAACAGGTTGCCATTATGACGCTTCCAAAACAGAAGGGCCAAAATGTACTTTGAAAAAGGAATTAAAAGCTCAACTAGACAAAGCAAACCAAGAAACAGGAGGGAAAGATGGCAAACCCACAAACACCACAGGAAACAATTCGTTTCTTATTAGTAAGGCCCCTCTTTGGCTTGCGGTTTTGCTTTTTTAAATTCCCCCCTCTTTTTTTCTTAAAGAAAATTTTGCTAAAATTTTTTGCTACTTGAAAAAACTTTCTGATATATTTTAACACGTAAGTTACCCG

>mVSG8 | length=1553 | **Tbb1125VSG-385**

TATTAGAACAGTTTCTGTACTATATTGCAAGGTAGAAGACTAATGACTGAACTCACCACAGGCAAACTATTCCTTTTCTTGCTGCTCATTGCGTGCTGTGTCAGGTCCCAGGCGGTTCATGACACGAAGAAGCAGATTAAAAACGCTTGCGACAGCAGCGACCACATGAAGATCATCGCCACTCGGCTAGCCGGTGCGCTGGCCAGCCAAGCGGCGGAAATACGCACAGCCGAACTCAAGCAGGCAAAGTTAGCAGCCGCAGCCAGCGCGCAAAGCCGAACAGTTGCTCAACGGTTGGCCCCGGTTCTAGCAGCATACGGAACAGAGCTAAGCAAAGCGAAACAGGCGTTGTGGAGTGCTTTGCCGGCAGTCATAGCAGGCGCATCAGCAGCTTCTCAGCTGGCAGCAACGCAGTCGATGATAGCCGACGTCGCCAAGGTAGCGCTCGACGACGCTAACACACTGGCGGCGAACGGTTTTTTTACCGCCAATCAAGGCAAGCAATTAATACCGAAAACGAAGGCGACCAAGGATTCGGCCTGCTCGGCAACAACTACCGCCACCCGCGACAACGAAGATCGCGAACGCGACTACAGCCAAACAATAACGGCGCCGTTTTACTTTTTAACCGAGTCAGACGCCACAACTCAAATACAGGCGGGCCCGAGGCTATGTGGCGACGACGCGGCAGGTCAAGCGCCGTGCAGCGACGCAACTAACGCAGTAAACGGAGCCAACATAGGTATAAAAGGAGGACCGTTGTTGCAAACCAAGGCGGCGTCATATAGCAGGAGGATAGGCGACGGCAGCTACACAACAGCCTCTATACAAGCAGCCAACGCCATTCCCACAAAAGATTACGTCGAAGAGCAGCTGAGCAACATCAAAACCGCAGAAGATGCAATCAAAGCGTTAACATTCAAGGAGGCTTCAATAGGCAGCCACAACCTAAAGCAAAATCCAACATTCACGGCTGCGGTCGCAAAGATAATGCTTAAGCAAGACAAGCCGCCAACAACAGGCGCAGATCTAAACGCACTAACAGCAGTCATCAGGCAGCAATACGGCGCAGATGAAGGCGAGTACGCAGCAAAGGTTTGGAAGGTAGTTGATAGTACAACGGCTCAACACGCCGTCGATGGTCACGCCGCAACCGCCACGATAGGACAACTACCTTCACAACCGGCGCTAGCGATGTTGACAGCATATAACCTGGCTGGCAAGCCGCCAGCTTCACCAGTTTGTGTCGATGAGTCGCAACAAGTAGTAGATTCCAAAAAAACAAAAGAATGCAAGGAAGAAAAAGACAAGGATAAATGCAACGAAAAGAATGGGTGTGAATTTAAAGATGGAAAGTGTGAAGCTAGAGTAACAGAAACAGAAGCAGGAAAAACGGGTACTCAAAACACCACAGGAAGCAATTCTTTTGTCATTAACAAGGCCCCTCTTTTGCTTGCAGTTTTGATTTTTTAATCCCCCTCCGTAAAGAACTTTGCTACTTGAAAACCTTTCTGATATATTTTAACACCTAAAAAATCAGCCGAAAAAAAAAA

**Predicted proteins**

mVSG1/2

MPKTSRLCSLGFFVLALLAESSRTATTYTICSTPCKCSKRLGKASTFYAQKFETNVGKLVKMQTDLTKLLLAATAADVATAKTALPTLAAAGKVIQDCQAAVTRQLAALKTGLPKIANASAKLAALARRQATKTTVKLTPKGGTSNYKDTSFANPPVGITSDDSCGHETGDGETDYDDNEADEKNAILEPQEYHTVTVTCRSTGTTNCNSAGQTQDDGYIQFELTSAIKEETSKPTTRWNAANTDSDVIIHGAVNVTQGTKEPTEAALKELKQAAQNTACDKKLTEYATVSASPLFRRQAIRSLLNQDADAQDFTTPPDKLTGALTAAYGAGGMDYKKKLWEAIDNLKPAITKNKERAELDIKENTPLEQLTEALARQIGEANSKASQTTKNNKNANDPSKSDAADKKEEKKDGDQKDEECKATEEGKCDKTKCDWNAEKKECKVKEGAAVISAVIKAPLLLAFLLF

mVSG3

MRGKVYALALLLATHDSAEATPNGIKAGAINRLCSLSDNLKAYTTYTKTAIATALTAVNDVQTVKQKLILLVLHTKKLPTQNAPLILSFMERTISQAILDLKQNAPTAVLAAAAAALSSGRIDEYTQLLYNANTNQPGLDKYCVAKDTGASKADPTDLGSCISEGKWKTKITDVPNTVAPSYATSVPNQADAQSVTAHTGQDNGCMLLSSASGDGFGVTSHAGKDILVMGGIFKLGTTALAASPFKDVSATAAPNTPLEQLKAIDHSQFPKIAGPAVTILKKLASLGKGADFDIPDQTIQKADYGLGAEGTLQIASSDFKKINTALQQFEASASDGLQRLLTQLPSALLSHAAMNCTVQSSAGGEPKTTADRSDCNSHAEEEACRKANCNFDGSKKPKCFAKPSENQSDKQDGESRKKEQNATGSNSFTIKKAPLLLAFLLF

mVSG4

MATLRAVLSTTLICFVFSTARGAKETLDATNHIKPICKTSGQLKALELYIASKLETGRQAANGLSARRAPFTLYAMSTESQADKTNCRLVATYIEKKAASVLTDMAETFVAALKATGAAHYAAGVIDGGIETLASESSSGEHCITKADGTTKADKSDLAGCGTADEDYTQYKNDWETKTAGQTLSAITTEADITVGGSSNKCALWTGASNLHSQTAQTVVWAGGTIAFAAGSDDTVQRRQITKSNTPKIFPAIKLIDDLRNKISTASWTDPMEAFFSKSGDDSAEALALATEISNSLKNKPTDAEPNVSSVKETLQAVKKASKQDPIKSQLTKTPLQKQAEIQLTINTCPTCEIKLAAATSSTTSASTGTAADCKSKKGDECKGECELDGESCKPKKQAEEVKKENEKTTNATGSNSFVINKAPLLLAFLLF

mVSG5

MIGKAFIILSLLNELPSPTAAQAAEGGALGKDVWLPLAKFTATAAKIPGRAAKLLQDRSAQIVNLMKLQVQADICLNKAASEVSALGWQALAVAIAADIGSLQSLQQQRSEEAIAAAAAAEFARGHAAEFFKVAAAVQSAANSGCLTTNNKGGAAGSVINGFSTLGTAEQPAIGATSTAHVGDDITAITTTGFSDLAATDGIRTDSLTADTNCVLFKGGSAGPLTTANFGQSIPFAGGYLTRNPTANTASSADGTDFVTNPGDGKIAGIKVYRDAHAAAAKIRTAATFGSSFTDFKKLDQAKKSVHLRTAVKNIILGKPDGSVADLSDEIDTKINQVFGEDQATFHSRFWDQLTKVKVEKAASGQDETTLDAITSFAALSRARTYYSTKVIKGLRDKISSLEIKNSKTEVKVTDADCNKHQSKDKCAAPCKWNENTTDINKKCSLDPVKATEQQAAQTAGAGEGAAGTTTDKCKDKKKDDCKSPDCKWEGETCKDSSILLNKQFALMVSAAFVALLF

mVSG6

MRHTGISTLCVFLLALTNSRAANHHPLKLNAWKPACELAGQLKSVTSVALTTIHRHQQESDASRKTAYAVLLYAAANKDAAADIAAEGLATALFQHSEAQQATAASSMRIGLTATQSAAQLSGAITATIDFLAAASHDAVYCLGNSAGSADDAANRRRYGCSYALAEPTADAQLLRSPAVDSKGFAELSDVVDGSTVDSSAQDKCIVTEHGSDHTKLLNDAGHNNKLVGGLFDFTTGRQAKRSGYATVAPKGERATQEVQATAFHDYAELRAAYKSKATTDPVELIAAVAASDYLKPAVAEYLASTSGATKPTNHEAMASAIIDKHYKNNGDGIKNLWERIKNTQVLDITKANGIKTKIVQIGTASTLLRTLALYNREALAKISKLEAAAAEKPTAKTTKEKAPTEEQCMHHETTGTCQNEGCEFDETKTPRCFPKPSETKEEKKDKQDGKTNTTANDSIVINKAPLLLAFFLF

mVSG7

MKQHLVFAAAAAIISLAPAAHVEAAAGDALNHAAWSKLCDITRDLDNLPSNELASIAAEQVNVGDLTKLQQQLALYRVLNTDKAATTAEQVFATFLSRKISGAAISETKLKEALQSTADAAFLHGQLAEWLATASSIGGNTAGCLGAAGGADTADKATITQAPYACKLTTQMASAKLTTPASIDANGYTGFTAIPNVETSNAVTQKKCAYSQHGASGLGGAQDTTTSIAFAGGAFILTDNALTRSNWAATSQAGTLPHEKAFKALKRQHKSLPKANYTKITDLKQDSDFRSAAIAIIYGDQDSSQLENKLTDLFGASDNSFEEKFWKNVREAKIDGGKFGAKEATTIQALEDPIKLSKAFYYYAKQGTEQLKTLEARAAATNDLPKPTEELCNAKNDEPKACNEATGCHYDASKTEGPKCTLKKELKAQLDKANQETGGKDGKPTNTTGNNSFLISKAPLWLAVLLF

mVSG8

MTELTTGKLFLFLLLIACCVRSQAVHDTKKQIKNACDSSDHMKIIATRLAGALASQAAEIRTAELKQAKLAAAASAQSRTVAQRLAPVLAAYGTELSKAKQALWSALPAVIAGASAASQLAATQSMIADVAKVALDDANTLAANGFFTANQGKQLIPKTKATKDSACSATTTATRDNEDRERDYSQTITAPFYFLTESDATTQIQAGPRLCGDDAAGQAPCSDATNAVNGANIGIKGGPLLQTKAASYSRRIGDGSYTTASIQAANAIPTKDYVEEQLSNIKTAEDAIKALTFKEASIGSHNLKQNPTFTAAVAKIMLKQDKPPTTGADLNALTAVIRQQYGADEGEYAAKVWKVVDSTTAQHAVDGHAATATIGQLPSQPALAMLTAYNLAGKPPASPVCVDESQQVVDSKKTKECKEEKDKDKCNEKNGCEFKDGKCEARVTETEAGKTGTQNTTGSNSFVINKAPLLLAVLIF
