# Supplementary material for: Developmental changes and metabolic reprogramming during establishment of infection and progression of Trypanosoma brucei brucei through its insect host
Source: PLoS Negl Trop Dis. 2021 Sep 20;15(9):e0009504. doi: 10.1371/journal.pntd.0009504 (PMC8483307; doi:10.1371/journal.pntd.0009504)
Supplement: Attachment — Submitted filename: PLOS_NTD_reviewer_response.docx [file pntd.0009504.s013.docx]

Reviewer's Responses to Questions

Dear Dan,

Thank you for providing the reviewers’ comments on our manuscript. We are happy that all three of them consider that it provides a valuable dataset and we are grateful for the constructive suggestions. For simplicity, our answers are interpolated in the text.

We hope these revisions now make the manuscript acceptable for publication.

Best regards,

Isabel Roditi

**Reviewer #1**: I only have a few minor points that could be addressed by the authors.

1. Lanes 449-451. The PFK-2 (6-phosphofructo-2-kinase)/FBPase-2 (fructose-2,6-bisphosphatase) enzyme produces or dephosphorylates fructose 2,6-bisphosphate (Fru-2,6-P2), a signal molecule that controls glycolysis and gluconeogenesis (Rider et al, 2004, Biochem J). Consequently, PFK-2/FBPase-2 is not involved in the production of the glycolytic intermediate fructose 1,6-bisphosphate (Fru-1,6-P2). In trypanosomes, Fru-2,6-P2 has been described as the main allosteric activator of pyruvate kinase and is involved in the control of the metabolic flux, rather than being a metabolic intermediate of glycolysis/gluconeogenesis. It seems that the authors have confused PFK-2/FBPase-2, and PFK and FBPase. This should be clarified in the text.

We thank the reviewer for pointing out this mistake. We address this point in lines 459-463.

2. Lane 414. "Table S4" should be replaced by "Table S5"

This has been corrected in line 411.

3. Figures 6 and 7. It would be relevant and very convenient for the readers to add, after the gene number, the name of the gene when known, as well as the function, in particular for amino acid carriers, as the author did for Figure 8.

We have done this as far as possible and now provide updated versions of these figures.

Reviewer #2: See assembled review comments

Reviewer #3: Minor revision.

--------------------

Summary and General Comments

Use this section to provide overall comments, discuss strengths/weaknesses of the study, novelty, significance, general execution and scholarship. You may also include additional comments for the author, including concerns about dual publication, research ethics, or publication ethics. If requesting major revision, please articulate the new experiments that are needed.

**Reviewer #1**: This combination of genomic (DNA) and transcriptomic (RNA) data provides new insights into trypanosome biology and will be useful resources for the trypanosome research community, including identification of transcripts obtained by single cell sequencing technologies.

**Reviewer #2**: This manuscript combines two components. Firstly, it provides a genome resource for T. brucei EATRO 11125 (‘AnTat’s’) that will be of value to the trypanosome research community that focus on developmental events and the in vivo biology of the parasite. Secondly, it provides a transcriptome description of several developmental stages as the parasite progresses through the tsetse fly, adding to existing datasets derived from salivary gland parasites, as well as very detailed transcriptome and proteomic studies of parasite development induced artificially in culture through RBP6 overexpression. Again, this provides a useful resource for the research community.

Overall, the datasets are of value and will be valuable when incorporated into TriTryp DB where they can add to the information available for researchers when understanding gene function in the parasite. I will break my comments into those concerned with the genome analysis and those concerned with the transcriptome analyses.

Note that these quite extensive notes and requests for clarification or technical amendment are to improve the utility and accessibility of the data and to reassure readers and provide maximal value to them. Overall, I consider the data provided will be a valuable resource that should be published.

1. Genome analysis

Although their methodology is likely to have produced a usable genome assembly, especially due to the use of the companion pseudochromosome assignment tool, I would like to see a few more quality control and assembly details before it is published. For example;

• I was unclear how much data was produced with the PacBio DNA run? What was the predicted genome coverage?

We obtained 321,316 CCS reads (1.35 x 10^9^ bases). This amounted to 37.4-fold coverage for the 11 megabase chromosomes; extended to the mini- and intermediate chromosomes, coverage was 21-fold.

• It doesn’t appear error correction for the Pacbio reads was carried out by illumina- is this the case?

This is correct. The PacBio HIFI reads (CCS reads) were extremely accurate. We compared them with sequences derived by Sanger sequencing (e.g. the procyclin loci in Genbank) and Illumina seq (de novo assembly) and obtained matches of 100%.

• Some basic assembly statistics should be provided (e.g., N50, contig number, GC content, numN50, mean contig size, max and min length of contigs) and this should be compared to a reference genome (e.g., Tb927).

We have provided this information as an extra tab in Table S1.

• A BUSCO assessment is needed for the genome assembly – this provides an unbiased assessment of the assembly - completed, fragmented and/or duplicated.

This has been done – the analysis is provided as a separate tab in Table S1.

• The coverage of each contig should be provided. I realise the number of reads mapping to each contig is provided, but this doesn’t summarise the coverage of each contig. 842 contigs seems to be very high and a quick examination of Table S1 shows there seems to be several contigs with very few reads mapping (often only 1 read!). I wonder if the assembly should be collapsed to remove duplication?

Contig coverage is provided in Table S1. CCS read extraction removed any PCR duplicates.

• Although cultured cells were used for raw genome data, levels of contaminating DNA are best assessed via BLAST searches of each contig in case unrelated DNA has crept in during isolation/library preparation and therefore assembly.

All contigs were subjected to BLAST searches as recommended. No contaminants were found. In addition, NCBI also screened all contigs before releasing them.

• More detail could be provided when describing the assignment of contigs to chromosomes and for those resulting contigs which are suggested to derive from mini/intermediate chromosomes. For example, companion does assign contigs to the 11 megabase chromosomes but it is unclear which contigs have been mapped to these 11 chromosomes. It is stated that “The remaining contigs (28.1 Mb in total) could not be assigned to the megabase chromosomes. Of these, 73 contigs contained 177 bp repeats, which are characteristic of mini- and intermediate chromosomes (38), and 98 contained telomeric repeats. Many of these contigs were close to the expected sizes of mini- and intermediate chromosomes. Further analysis revealed that the majority of them encoded a single VSG at the end of the contig; few encoded more than one VSG.”

Table S1 contains an extra tab providing this information.

Based on this some further clarity is needed:

 How many contigs were remaining after the assignment to megabase chromosomes?

685 contigs were not assigned to the megabase chromosomes.

 Please quantify the statement about many contigs being of the expected mini/intermediate chromosome size (size and number of contigs).

191 contigs were identified as minichromosomes on the basis of NCBI blast hits. An extra column has been added in Table S1. Based on their sizes (ranging from 4.7 – 525 kb), some of these are likely to be intermediate chromosomes.

• It would be valuable to provide the alignment of contigs derived from AnTat to Tb927. This figure should be produced by default during the companion pipeline and this would show any genomic regions with potential deletions/ expansions in Antat, which is valuable information.

Thank you for pointing this out. This information has now been included as Supplemental file 1. Other supplemental files have been renumbered accordingly.

• The authors go onto validate their assembly by looking at known regions. They note that “In addition, the assembly of chromosome 10 confirmed the previously described organisation of EP1 and EP2 procyclin and downstream procyclin associated genes (PAGs) 1, 2, 4 and 5 on one copy of the chromosome and a PAG1/2 fusion, and no PAG5 on the second copy (40).”

 Are both copies of chromosome 10 included- I understood this was a haploid assembly?

It is a haploid assembly, with the closest version to TREU927. The contig containing the second version of this region (Contig 6; 23 kb) is marked in Table S1.

 BUSCO could provide a more accurate and unbiased approach to this this assessment.

We have performed this analysis. The 927 and EATRO1125 genomes give very similar outputs. This information is included as an extra tab in Table S1.

• a k-mer based assessment of their raw data could be run to estimate the predicted genome size before assembly. This would give a good idea whether duplication in the assembly is present, or if it is simply the mini/ intermediate chromosomes that have almost doubled the size of the assembly in comparison to Tb927

We used GenomeScope and obtained a predicted genome size of 33Mb, which is in reasonable agreement with the combined sizes of the 11 megabase chromosomes. Information on the GenomeScope Github page suggests that the (unknown) ploidy of the intermediate and minichromosomes might be an issue. To quote:

“GenomeScope is only appropriate for diploid genomes. In principle the model could be extend to higher levels of polyploidy by extending the model to consider additional peaks in the k-mer profile, but this is not currently supported. GenomeScope also does not support genomes that have uneven copy number of their chromosomes, such as aneuploid cancer genomes or even unequal numbers of sex chromosomes. In these scenarios the reported heterozygosity rate will represent the fraction of bases that are haploid (copy number 1) versus diploid (copy number 2) as well as any heterozygous positions in the other chromosomes.”

• They could also run purge_dups (https://github.com/dfguan/purge_dups) to remove potential duplication caused by heterozygous regions of the genome.

BUSCO identifies 8 contigs, one of which is the second version of the procyclin/PAG locus on chromosome 10. We have retained these as they are likely to be regions of heterozygosity in the genome.

2. Annotation

• Annotation: What settings were used with companion? There are many settings to choose from and the setting used should be stated. For example, in ‘Step 6: Advanced settings of companion’: changing the sensitivity of Augustus will drastically alter the number of genes annotated. Please clarify.

Default settings were used. This has been clarified in the text (line 158).

• Was the transcriptome data used during the companion annotation run?

Yes, it was – this has been added (lines 158-9).

• By running BUSCO on the companion protein file, in protein mode, it would also be possible to show how successful the annotation was.

This was performed and is presented as an extra tab in Supplemental Table S1.

3. Transcriptome assembly

Although the use of the 927 genome for transcriptome assignment was justified to allow comparison with earlier datasets, this seems a strange decision given the effort put in to deriving the AnTat genome. Further, the manuscript does not directly compare the current datasets with results from other labs using 927 except in the case of some specific gene sets- although I think this would be useful (see below).

Most papers reference gene IDs from TREU927. This makes it easier for comparisons. We already used a dataset from the Matthews lab (mouse-derived slender and stumpy forms). We now elaborate on other datasets in the Discussion (see point 4). However, the main impetus behind the genome assembly was to obtain accurate untranslated and non-coding regions, where there is greater variation.

I wonder what was the overall mapping rate of RNA reads to the new Antat pseudo-assembly.

We have included an extra tab (mapping comparison) in Table S1. For bloodstream forms the rate is 98.9% for EATRO1125 versus 94.57% for TREU927. This is largely due to the VSG repertoire. Importantly, for ChIP-seq, the rate is 95.23% for EATRO 1125 versus 77.58% for TREU927.

4. RNA seq analysis

The dataset generated is valuable and provides a good resource to explore transcripts expressed by the parasites as they traverse compartments within the tsetse fly. It is largely descriptive however and I feel an opportunity has been missed to compare the in vivo data with the impressive and high-quality transcriptome, proteome (and metabolome) datasets generated by the Zikova lab recently using RBP6 overexpression (Doleželová et al, PLOS Biology 2020; https://doi.org/10.1371/journal.pbio.3000741). The extent to which the datasets correlate would be very valuable to the community and allow better assessment of the utility of the in vitro model which is now being used extensively by several laboratories. Similarly direct comparison with the single cell data (Vigneron et al, PNAS 2020) and RNA seq data from infected tsetse salivary glands (Savage et al., PLOS One 2016) would be valuable.

Currently the figures summarising the expression profiles and datasets are quite limited, comprising heatmaps for different gene families and a non-quantitative summary figure of metabolism. I would hope a more detailed and intuitive analysis could be presented of the respective comparison of the current datasets with other datasets at different timepoints or with different experimental systems. This should be feasible via PCA plots for example to provide an assessment of the level of correspondence between, for example, published in vitro and in vivo models for some developmental stages in the fly, or single cell RNA data for the salivary gland and the RNAseq data provided here.

We have performed a principle component analysis using the data from the Zikova lab (see below). The datasets correlate very little, but this is not entirely surprising for two reasons: i) the RBP6-overexpressors were cultured in medium lacking glucose, and ii) in culture there is no separation of life-cycle stages that occur in separate tissues in the fly.

The vivo datasets from Savage et al. are based on a single, very late time point (day 40) and sequencing reads were much shorter, precluding unambiguous assignments of closely related genes. There was also 5’ bias in their sequences.

The Vigneron dataset is now dealt with in the text (lines 417-426) and an additional figure (Figure 6).

The details of the changes of various transcripts were often quite descriptive and transcripts highlighted without a clear indication of why these had been selected for description. For example, ““Several transcripts for metabolic enzymes peaked on D7, including fructose-1,6-bisphosphatase and the tricarboxylic acid (TCA) cycle enzymes citrate synthase, isocitrate dehydrogenase and aconitase (Figure 4; Table S3). Transcripts for a proline/alanine transporter from the AAT7-B family (Tb927.8.7640) (17) and delta-1-pyrroline-5-carboxylate dehydrogenase also peaked on D7.” Were these changes significant? What selection criteria were used for highlighting changes?

There were two criteria for selection. First, statistical significance. We only discussed transcripts that were significant in DESeq2. Another criterion was trypanosome biology: there are controversies about glucose metabolism in flies. This is also relevant in the context of recent findings by Villafraz et al (PLOS Pathogens, 2021) and our lab (Shaw et al., https://doi.org/10.1101/2021.01.01.424252).

Table 1: please add LogFC and significance to the Table

This has been done.

With respect to Figures 6,7 and 8. These figures are missing a scale for the colours used in the heatmap. Also have the genes and samples been clustered during plotting?

Colour scales are included. The genes were clustered. If the samples are clustered as well, each figure starts at a different point in the life cycle, which is confusing.

Some technical points that would be good to clarify with respect to the transcriptome analysis

• I found the methods for the transcriptome section slightly outdated. They deviate from the methods suggested by the DEseq2 vignette –

There was no deviation, we used default parameters:

<http://www.bioconductor.org/packages/release/bioc/vignettes/DESeq2/inst/doc/DESeq2.html#differential-expression-analysis>.

Columns B and C and J-M were added manually. We have relabelled B and C to make it clear that they are RPM.

• Please confirm the type of data that was passed to DESeq2 – raw or RPM values? – Deseq2 requires untransformed data.

Raw values (untransformed data) were used.

• Which version of Trinity was used for the de novo transcriptome assembly?

Trinity-v2.5.1. This has been incorporated into the manuscript (line 202).

• A BUSCO completeness assessment should be performed on the transcriptome assembly

This has been done and is included as an extra tab (assembly statistics) in Table S1.

• “When we compared this annotated file with the single cell RNA sequencing data generated by Vigneron and co-workers (41), we found an excellent correlation between their 3' reads and the mapped ends of transcripts”. How is an excellent correlation defined in this context?

This was an unfortunate choice of word. “Correlation” has been replaced by “agreement”. In addition, a supplemental figure (S1) is provided.

• RPM is used for comparisons of transcript levels, but this does not account for gene length. TPM is more suitable I believe.

We compare transcript abundance between stages so length will not play a role.

• “Moreover, the percentage of reads assigned confidently to the transcriptome increased from 41% to 48.5%”. Please specify what is meant by a confident assignment. Is a mapping quality cut off used? I don’t believe this was specified in the methods section.

Assignment was performed with Cellranger version 6.0.0 using default parameters. This is included in the text (line 259). We have removed the word “confidently”.

Reviewer #3: This is a descriptive but very useful article describing (i) the genome of a pleomorphic strain of Trypanosoma brucei that a growing number of labs is using (EATRO1125), and (ii) the transcriptome of parasites colonising different organs of the Tsetse fly (the insect host). Having the genome of EATRO1125 will increase the accuracy of future genome-wide analysis and it will improve the efficiency of editing using CRIPSR/CAS9 technologies. The comparison of the transcriptome of in vivo parasites from midgut (several time points), proventriculus and salivary gland showed that there is a constant remodeling of gene expression, including of metabolic proteins, surface proteins and sensing factors (such adenylate cyclases). The transcriptome studies also allowed the identification of ~9300 genes, in which the 5’ and 3’UTRS were mapped, which is another useful tool for the community.

The experimental work is solid and the conclusions are sustained by the results obtained. I have a few minor suggestions, most of which aim to improve the clarity of figures and supplementary materials.

Minor comments

1. The pairwise comparisons described in Figure 3 and Table S3 are useful to identify the genes differentially expressed between two stages. However, an additional figure is missing to show the global patterns of transcript changes: to visually see which stage is more different, how many genes contribute to that difference, the level of up/down regulation. This could be achieved with a heat-map of all genes that are differentially expressed in at least one of the 7 time-points collected from the tsetse (similar to the heat-map of Figures 6-8); along with this global analysis, the authors could ask if there are co-regulated genes with similar patterns of gene expression in some or all stages in the Tsetse. This co-expression may result from a common regulatory mechanism, which could depend on conserved elements in 5’ or 3’UTR (if they exist). These global analysis could actually be part of Figure 1, after the PCA plot.

We have provided the global analysis as Figure 1B although we don’t find it that informative.

2. The paper should include a figure about the genome, showing a schematic of megabase, intermediate and mini-chromosomes, their sizes and numbers. Authors could also consider adding a table displaying some of the data described in the text, such as saying how many contigs, how many genes identified, etc.

There aren’t precise numbers for these as the reference TREU927 genome is missing the ends of chromosomes and provides no information on the intermediate and minichromosomes. It is not even clear what their ploidy is. Table S1 includes the size of each contig.

3. For those readers less familiar with Tsetse life cycle stages, I suggest including a cartoon of a tsetse indicating the midgut, proventriculus and salivary glands.

We have included this as a supplemental figure (Figure S1).

4. In data presented in Figure 3A/B, the authors should indicate the number of differentially expressed genes in the text and perhaps even in the top of each volcano plot. I know this information is available in Table S3, but indicating it in the figure would help the reader.

These numbers have been added as requested.

5. Line 414. I believe the data about RHS7 is in Table S5, not Table S4.

This has been corrected in line 411.

6. In *T. brucei*, VSG genes are classified as a- or b-type. To which type do metacyclic VSG identified in this study belong ? Is there any sequence similarity to metacyclic VSG genes in T. congolense? I suspect not.

Since we are not experts in this, we enlisted the help of a colleague (Mark Carrington). He classified most of them as A2-type, with the exception of mVSG1/2 (type C4) and mVSG5 (A1-type). A BLAST analysis did not identify sequence similarity to *T. congolense* mVSGs.

7. Given that Table 1 depicts surface protein transcripts, shouldn’t GCS1/HAP2 be included?

The reviewer is right, it is a membrane protein, but it didn’t fall into any of the categories in the Table. We have changed the heading to one of the columns and have now included it.

8. In Figure 8, the authors should label the clusters of genes encoding for proteins involved in Glycolysis, TCA, etc. Otherwise, it is not really possible to visually interpret the heat map in terms of changes in metabolic functions. This

This is why we included Fig 10 as a visual summary. Glycolytic enzymes are in the glycosome; TCA enzymes are in the mitochondrion.

9. Supplementary tables – Could the author add the title of the table as line 1? This will help go through the multiple supplementary files and more clearly identify each file.

These have been modified as requested.

--------------------

Figure Files:

While revising your submission, please upload your figure files to the Preflight Analysis and Conversion Engine (PACE) digital diagnostic tool, https://pacev2.apexcovantage.com. PACE helps ensure that figures meet PLOS requirements. To use PACE, you must first register as a user. Then, login and navigate to the UPLOAD tab, where you will find detailed instructions on how to use the tool. If you encounter any issues or have any questions when using PACE, please email us at figures@plos.org.

Data Requirements:

Please note that, as a condition of publication, PLOS' data policy requires that you make available all data used to draw the conclusions outlined in your manuscript. Data must be deposited in an appropriate repository, included within the body of the manuscript, or uploaded as supporting information. This includes all numerical values that were used to generate graphs, histograms etc.. For an example see here: http://www.plosbiology.org/article/info%3Adoi%2F10.1371%2Fjournal.pbio.1001908#s5.

Reproducibility:

To enhance the reproducibility of your results, we recommend that you deposit your laboratory protocols in protocols.io, where a protocol can be assigned its own identifier (DOI) such that it can be cited independently in the future. Additionally, PLOS ONE offers an option to publish peer-reviewed clinical study protocols. Read more information on sharing protocols at https://plos.org/protocols?utm_medium=editorial-email&utm_source=authorletters&utm_campaign=protocols

__________________________________________________

In compliance with data protection regulations, you may request that we remove your personal registration details at any time. (Use the following URL: https://www.editorialmanager.com/pntd/login.asp?a=r). Please contact the publication office if you have any questions.
