# Supplementary material for: Developmental changes and metabolic reprogramming during establishment of infection and progression of Trypanosoma brucei brucei through its insect host
Source: PLoS Negl Trop Dis. 2021 Sep 20;15(9):e0009504. doi: 10.1371/journal.pntd.0009504 (PMC8483307; doi:10.1371/journal.pntd.0009504)
Supplement: Attachment — Submitted filename: Response_to_resubmission.docx [file pntd.0009504.s014.docx]

Dear Dan,

We are happy that reviewers 1 and 3 were satisfied with the changes.

We have addressed all the points raised by reviewer 2 (interpolated below) and hope that the manuscript is now acceptable for publication.

In the meantime, we modified our GFF file according to recommendations by TritrypDB; we have uploaded this version together with the revised manuscript.

Best regards,

Isabel

Reviewer #2: The authors have done a good job at rebutting earlier reviewer comments, although a slightly a less good job of revising the manuscript to reflect these rebuttals. I suggest some of the comments in the rebuttal make it into the manuscript with, where appropriate, some discussion.

For example:

The BUSCO values are now included for the genome assembly and included in the Supplementary Table but there is no discussion of this in the main manuscript text. Please include comments in the main manuscript. Please also specify the version of BUSCO used and the database. Indeed, please go through the manuscript to ensure all analysis packages state the version used as this can have an important impact on their outputs and so the reproducibility of the study.

The BUSCO version is: 5.0.0. The lineage dataset is: eukaryota_odb10 (Creation date: 2020-09-10, number of species: 70, number of BUSCOs: 255). We have included this information in the results section (line numbers 246-250).

The coverage data is now included though significant numbers of contigs have a coverage of only 1 or 2, which greatly reduces confidence. Please include a comment in the main manuscript- not simply the Table values.

Only 68 contigs have the coverage ≤2, and together these amounted to less than 1 Mb, so less than 1.5%. We really don’t think that these merit a special mention in the main text. The long PacBio HiFi reads are built from several raw reads, so this coverage is not comparable to coverage obtained by Illumina sequencing.

The newly included analysis of genome size indicates some expansion of the AnTat genome by around 3Mb. Please elaborate on the basis of this expansion in the manuscript text.

As Supplemental file S1 shows, there are insertions in all 11 megabase chromosomes compared to TREU927. This is mentioned in line 234.

Comparisons with previously published datasets are now included via a PCA map in the rebuttal (which should be included as a supplementary figure). There is quite a large difference from, particularly, the datasets generated via RBP6 overexpression. Similarly the scRNA analysis (Figure 6 now includes this). Good justification for these differences is provided in the rebuttal but there is not an equivalent discussion in the main manuscript I think. I consider an expansion of text would be helpful to clarify why datasets between labs likely differ so that readers can best interpret the respective studies.

We have included the PCA map as Figure S2 as well as some new text (lines 440-445).
